# Earthworms Use Odor Cues to Locate and Feed on Microorganisms in Soil

**DOI:** 10.1371/journal.pone.0021927

**Published:** 2011-07-20

**Authors:** Lara Zirbes, Mark Mescher, Véronique Vrancken, Jean-Paul Wathelet, François J. Verheggen, Philippe Thonart, Eric Haubruge

**Affiliations:** 1 Functional and Evolutionary Entomology, Gembloux Agro-Bio Tech, University of Liege, Gembloux, Belgium; 2 Center for Chemical Ecology, Department of Entomology, Pennsylvania State University, State College, Pennsylvania, United States of America; 3 Laboratory of General and Organic Chemistry, Gembloux Agro-Bio Tech, University of Liege, Gembloux, Belgium; 4 Laboratory of Bioindustries, Gembloux Agro-Bio Tech, University of Liege, Gembloux, Belgium; New York State Health Department and University at Albany, United States of America

## Abstract

Earthworms are key components of temperate soil ecosystems but key aspects of their ecology remain unexamined. Here we elucidate the role of olfactory cues in earthworm attraction to food sources and document specific chemical cues that attract *Eisenia fetida* to the soil fungi *Geotrichum candidum*. Fungi and other microorganisms are major sources of volatile emissions in soil ecosystems as well as primary food sources for earthworms, suggesting the likelihood that earthworms might profitably use olfactory cues to guide foraging behavior. Moreover, previous studies have documented earthworm movement toward microbial food sources. But, the specific olfactory cues responsible for earthworm attraction have not previously been identified. Using olfactometer assays combined with chemical analyses (GC-MS), we documented the attraction of *E. fetida* individuals to filtrate derived from *G. candidum* colonies and to two individual compounds tested in isolation: ethyl pentanoate and ethyl hexanoate. Attraction at a distance was observed when barriers prevented the worms from reaching the target stimuli, confirming the role of volatile cues. These findings enhance our understanding of the mechanisms underlying key trophic interactions in soil ecosystems and have potential implications for the extraction and collection of earthworms in vermiculture and other applied activities.

## Introduction

Olfaction is a key sensory modality by which animals, and many other organisms, acquire information about the surrounding world [1], [2]. In addition to perceiving semiochemicals (i.e., pheromones and kairomones), which play key roles in many interactions within and between species [3], many organisms also detect and respond to general odorant cues deriving from biotic and abiotic features of their environments. Indeed, most organisms have specialized sensory, information processing, and behavioral mechanisms dedicated to detecting and reacting to chemicals present in the external environment [4], [5]. Among terrestrial invertebrates, the perception and use of olfactory cues by insects, and some other arthropods, has been extensively studied [2], [6], [7], [8] as has the ecological role of olfactory cues in interactions among insects and plants (e.g., [9], [10], [11], [12]) Previous work has also explored the detection of olfactory cues by nematodes which entails the activation of papilla and setae on the body surface that are connected to chemosensory neurons known as the AWA cells [1], [13] and the response of nematodes to various olfactory cues [14], [15], [16]. In contrast, olfaction by annelids remains poorly studied. Chemoreceptors have been identified on the prostonium and the buccal epithelium of earthworms [17] and have been shown to detect sucrose, glucose and quinine [18]. Recently, olfaction by earthworms has been suggested to be involved in the coordination of collective movement [19].

Previous studies exploring the feeding strategies of various earthworm species suggest that these animals exhibit orientation and movement toward particular food sources, including specific species of protozoa, bacteria, fungi and plants [20], [21], [22], [23]. Microorganisms are both major components of earthworm diets [20] and principle sources of volatile organic compound emissions in soil ecosystems [24], suggesting that olfaction may play a key role in earthworm foraging. Moreover, Bonkowski and Schaefer [25] reported that *Aporrectodae caliginosa* actively moved toward foraging sites exhibiting higher densities of protozoa and naked amobae. Soil fungi are particularly important food sources for earthworms, especially for epigeic species that consume litter typically colonized by fungi [26], [27], including *Geotrichum candidum*, *Mucor* sp., and *Aspergillus flavus* [28], [29]. Bonkowski et al. [28] conducted feeding choice assays to document the preferences of five earthworm species for a variety of soil fungi and reported a general pattern in which worms exhibited a preference for early successional species (e.g., *Fusarium nivale* and *Cladosporium cladosporioides*) that are presumably indicative of relatively new and nutrient rich organic resources. The factors underlying the observed preferences were not determined, though the investigators postulated that differences in the nutritional value of the fungi, or the presence of antibiotic compounds or other metabolites in or around the mycelia might be important.

Because soil fungi release volatile and non-volatile chemicals, as well as influencing the release of plant-derived compounds [30], [31], olfactory cues associated with the presence of fungi may be expected to play an important role in earthworm foraging for fungal food sources. However, previous studies have not explicitly addressed the role of olfaction or documented the specific cues responsible for orientation and attraction. Therefore, we explored the role of olfaction in the foraging of *E. fetida*, an epigeic earthworm species with economic significance for various industrial processes, on the soil fungus *G. candidum*, which is an important food source for this worm [29].

## Methods

### Eisenia fetida rearing

Earthworms (*Eisenia fetida*) provided by Ouroboros s.a. (Belgium) were reared in PVC boxes (42 cm long×30 cm wide×10 cm high) filled with universal compost DCM ® (De Ceuster Meststoffen s.a.,Grobbendonk, Belgium) composed of a mixture of brown peat, white peat, and lava. The compost was changed every two months and cocoons and hatchling earthworms were placed in new boxes with fresh compost. Boxes were maintained at 23±1°C. Only mature earthworms (with a clitellum) were used for our experiments.

### Culture of Geotrichum candidum

*Geotrichum candidum*, isolated from compost mixed with milky fermented product, was cultured in 100 ml of liquid medium 863 (2 g glucose; 1 g yeast extract; 1 g peptone) at 27±3°C during 42 h, and the culture was filtered with Pall Supor® (Whatman Ltd, England) - 450 membrane 47 mm–0.45 µm filter.

### Bioassays

#### Earthworm response to cues associated with Geotrichum candidum

**Figure 1:**
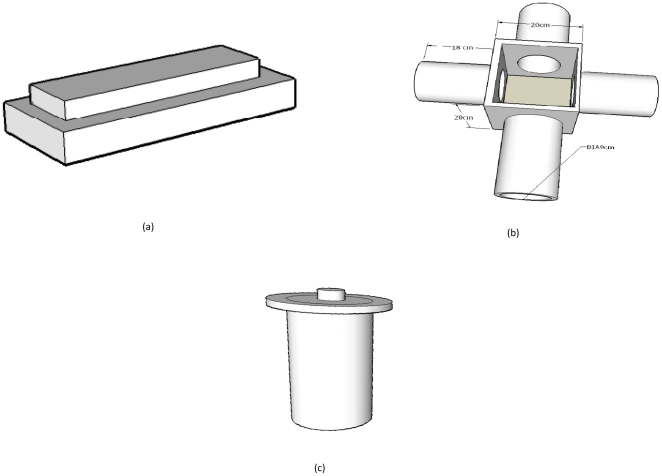
Experimental set-up. (a) Dual-box earthworm sampling device, (b) four-arm olfactometer (A = central chamber, B = (identical) arms), (c) vertical olfactometer.

A PVC box (Box #1: 56 cm×36 cm×8 cm) was filled with moist compost (76% humidity content; obtained by drying a 25 g sample of moist compost at 105°C for 48 h), and 200 earthworms (100 matures and 100 immatures) were placed randomly within it. A second box (Box #2: 37 cm×26 cm×9 cm) was placed on top of Box 1 (Fig. 1). Box 2 had 5 slots (30 cm long×0.5 cm wide) in its bottom and was also filled with moist compost (prepared as above) Filtrate (275 ml) from the *G. candidum* culture was then poured evenly across the surface of Box 2. Pairs of control boxes were similarly placed but received tap water instead of the fungal filtrate. After 120 h, the number of earthworms in each box was determined. Six repetitions were conducted with the *G. candidum* filtrate and three for the controls.

#### Four-arm olfactometer experiments

Earthworm behavior was more precisely observed in a below-ground olfactometer consisting of a central PVC chamber (20 cm×20 cm×10 cm) connected to four equidistantly space side arms (9 cm in diameter, 18 cm long) (Fig. 1b). For each experiment, the entire system was filled with moist compost (as above). Target stimuli (i.e., *G. candidum* filtrate or filter paper treated with individual compounds) were placed at the far end of one arm (selected randomly), while the three remaining arms acted as controls. Between repetitions, each piece of the olfactometer was cleaned with tap water and then with norvanol before being dried overnight at 70°C. For each stimulus tested, groups of earthworms varying in number/density from 5 to 160 individuals were introduced in the central chamber and allowed to make choices (the specific numbers used for each assay are listed in table 1). One day after release, the olfactometer was disassembled, the compost in each arm was placed in a separate container, and the number of earthworms was recorded. The specific stimuli assayed are presented in table 1. When testing effects of volatile cues on earthworm behavior, a circular metal screen was placed in the middle of each arm to prevent physical contact with the target. Four different doses of ethyl pentanoate and ethyl hexanoate (1 µl, 10 µl, 100 µl and 1000 µl) were evaluated (sample purity was 97% and 98%, respectively for the two esters). At least 10 replicates were conducted for each trial. A control with only compost in the four arms of the olfactometer was also employed in 3 replicates.

**Table 1:** Treatments employed in the four-arm olfactometer bioassays.

Experiment	Number of earthworms	Tested substances	Quantity of tested substance	Repetitions
Influence of *G. candidum*	20	G. candidum filtrate	25 ml	18
Influence of earthworms density	5	G. candidum filtrate	25 ml	18
	10	G. candidum filtrate	25 ml	18
	40	G. candidum filtrate	25 ml	18
	80	G. candidum filtrate	25 ml	18
	160	G. candidum filtrate	25 ml	18
Influence of identified volatile compounds	20	Ethyl acetate	1 ml	10
	20	Ethyl propionate	1 ml	10
	20	Ethyl pentanoate	1 ml	10
	20	Ethyl hexanoate	1 ml	10
	20	3-octanone	1 ml	10
	20	2-methylbutan-1-ol	1 ml	10
	20	3-methylbutna-1-ol	1 ml	10
	20	2-methylpropan-1-ol	1 ml	10

#### Vertical olfactometer experiments

A second, vertical olfactometer (Fig. 1c) was also employed to study earthworm responses to chemical cues over greater distances and to test the feasibility of using chemical cues to attract earthworms to the soil surface, as in vermicomposting. This olfactometer constituted PVC tubes 9 cm in diameter and either 25 cm, 40 cm or 105 cm long that were filled with moist compost (as above). Ten earthworms were placed at the bottom of the olfactometer and 25 ml of *G. candidum* culture filtrate was introduced at the top. After 24 h (for olfactometers having heights of 25 and 40 cm) and 65 h (for the olfactometer having a height of 105 cm), earthworms present in the top five centimeters of the olfactometer were counted. As a control, similar trials were conducted without filtrate for each olfactometer length. Each experiment was replicated 18 times.

### Sampling fungal volatiles

A 5 ml sample of *G. candidum* filtrate was placed in a glass vial with a septum (opening) in the lid. A 75 µm carboxen-polydiméthylsiloxane (CAR/PDMS) solid-phase micro-extraction (SPME) (Supelco) fiber was inserted through the septum and exposed for 30 min at 40°C. The compounds adsorbed on the fiber were analyzed by gas chromatography-mass spectrometry (GC-MS). The GC–MS system comprised a GC (5890 Serie II Plus, Hewlett Packard) linked to a quadrupole type mass selective detector (5989A, Hewlett Packard). The fiber was inserted manually into the injector port (240°C), desorbed, and the sample chromatographed on an apolar column (Factor four VF-5 ms, 30 m, 0.25 mm internal diameter, 0.25 µm film thickness, Varian). Helium at a constant pressure of 55 kPa was used for carrier gas flow. After fiber insertion, the column temperature started at 40°C during 30 sec, increased to 180°C at 5°C/min then to 240°C at 15°C/min followed by a final stage of 2 min at 240°C. Electron impact mass spectra were recorded over the range 30–350 *m*/*z* (Electron energy: 70 eV). Identifications were performed by Wiley 275 library searches and by comparison with the retention time of external standards. Three replicate samples were analyzed. Volatiles of filtrated culture medium (medium 863) were collected and analyzed as controls.

### Statistical analyses

A Chi-square Goodness-of-fit test (Minitab® v15.0, State College, Pennsylvania USA - α = 5%, 3 degree of freedom) was used to compare earthworms' distribution in each arm of the four-arm olfactometer to a theoretical distribution based on random preferences for each of the olfactometer arms. A one-way ANOVA (Minitab® v15.0, State College, Pennsylvania USA - α = 5%) was used to compare the numbers of earthworms in the top five centimeters of the vertical olfactometer in presence or absence of filtrate.

## Results

**Figure 2:**
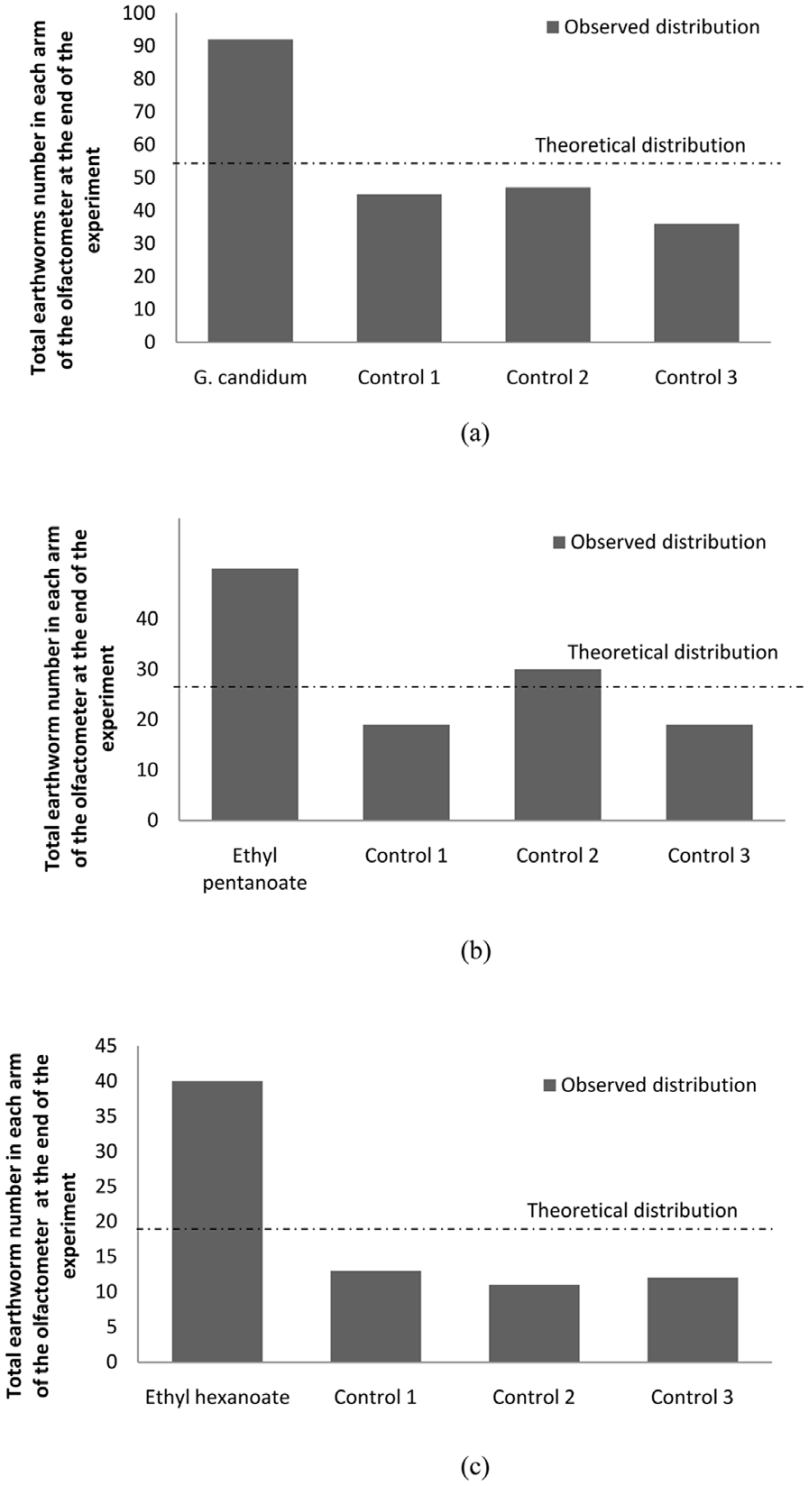
Earthworm behavior in four arm olfactometer. Observed and theoretical (i.e. random) distributions of earthworms in each arm of the four-arm olfactometer when one arm is treated with (a) *G. candidum* filtrate, (b) ethyl pentanoate (100% v/v), or (c) ethyl hexanoate (100% v/v). The distributions are compared by a Chi-square Goodness-of-fit test.

To determine whether *E. fetida* respond to olfactory cues associated with *G. candidum*, responses to fungal filtrate and specific compounds were examined in semi-natural conditions using pairs of stacked boxes. Significantly more earthworms were collected in target box when *G. candidum* filtrate was applied, 179±6.26 (mean ± SD) vs 87±23.31 (mean ± SD) when filtrate was absent (One-way ANOVA, p<0.001). Because of the long exposure time employed in this assay (120 h), it is possible that some fluid components of the filtrate, in addition to olfactory cues, may have percolated through box two and arrive in box one. Subsequent experiments, described below, more effectively test attraction to volatile cues alone. Initial experiments conducted with a four-arm olfactometer produced similar results, as significantly more earthworms were recovered from the olfactometer arms treated with *G. candidum* filtrate (Fig. 2a; Chi-square Goodness-of-fit test, χ^2^_3_ = 34.44, p<0.001). There was no apparent bias in the experimental set-up, as a fairly uniform distribution of earthworms across the 4 olfactometer arms was observed on controls where filtrate was not introduced. (Arm1: 7; arm2: 7, arm3: 3, arm4: 5; Chi-square Goodness-of-fit test, χ^2^_3_ = 2, p = 0.572). Similar earthworm attraction was observed across a range of population densities (Table 2). Moreover, in experiments conducted with three vertical olfactometers (25, 40 and 105 cm) earthworms reached the soil surface only when *G. candidum* filtrate was present (Table 3), demonstrating vertical attraction to olfactory cues over significant distances and thus the feasibility of collecting earthworms at soil surface with *G. candidum* filtrate.

**Table 2:** Influence of earthworm density on earthworm attraction in the four-arm olfactometer.

	Earthworms density					
	5 earthworms	10 earthworms	20 earthworms	40 earthworms	80 earthworms	160 earthworms
G. candidum	49	83	92	326	528	1536
Control 1	5	19	45	64	156	202
Control 2	11	18	47	60	127	169
Control 3	3	15	36	55	126	138
χ^2^	82.35	96.08	34.44	421.7	493.63	2742.69
p-value	<0.001	<0.001	<0.001	<0.001	<0.001	<0.001

Total earthworm number across all replicates is given in the table.

**Table 3:** Earthworm behavior in vertical olfactometer.

	Olfactometer height		
	25 cm	40 cm	105 cm
With *G. candidum* filtrate	8.3±0.3	6.9±0.2	6.1±0.4
Without *G. candidum* filtrate	1.3±0.3	1.3±0.3	0.4±0.1
p-value	<0.001	<0.001	<0.001

Numbers of earthworms (mean ± SD) collected in the top 5 cm of the vertical olfactometer in presence and in absence of G. candidum filtrate for each olfactometer arm length.

To determine the olfactory cues responsible for the observed attraction, volatiles from *G. candidum* filtrate were collected by SPME and analyzed by GC-MS. These analyses identified 18 molecules, of which 16 were specifically identified from filtrate of *G. candidum*: ethyl acetate, 2-methyl-1-propanol, ethyl propionate, 3-methyl-1-butanol, 2-methyl-1-butanol, ethyl 2-methylpropanoate, ethyl butanoate, ethyl but-2-enoate, ethyl 2-methylbutanoate, ethyl 3-methylbutanoate, ethyl pentanoate, ethyl 3-methylbut-2-enoate, ethyl 2-methylbut-2-enoate, 3-octanone, ethyl hexanoate, ethyl hex-2-enoate. Authentic standards of eight molecules that were commercially available were tested separately in the four-arm olfactometer. Two esters exhibited significant attraction of *E. fetida*: ethyl pentanoate (Chi-square Goodness-of-fit test, χ^2^_3_ = 0.3105, p<0.001) and ethyl hexanoate (Chi-square Goodness-of-fit test, χ^2^_3_ = 0.2173, p<0.001) (Fig. 2b and 2c).

The poor solubility of these molecules suggests that volatile cues, diffusing in compost, are likely attractants.

To confirm that volatile cues were responsible for attraction, attraction to the two esters was measured in the four-arm olfactometer using a metallic mesh to prevent the earthworms from contacting the odor source (thus ruling out contact cues). Significant attraction was observed for ethyl pentanoate at quantities above 10 µl and for ethyl hexanoate at quantities above 100 µl, and weak attraction was observed for both compounds even at levels as low as 1 µl (Table 4).

**Table 4:** Quantities of ethyl pentanoate and ethyl hexanoate tested and *E. fetida* responses to each.

Molecules	Quantity	Attraction	p-value
Ethyl pentanoate	1 µl	◊	0.032
	10 µl	◊	0.006
	100 µl	◊	<0.001
	1000 µl	◊	<0.001
Ethyl hexanoate	1 µl	◊	0.030
	10 µl	-	0.67
	100 µl	◊	<0.001
	1000 µl	◊	<0.001

- = no attraction, ◊ = earthworm attraction.

Note: Although p-values at 1 µl for both esters are significant (and in each case earthworms were overrepresented in the treatment arm relative to expectations based on a random distribution) attraction to the treatment arm was not significantly different than to at least one adjacent control arm. Instead earthworms were significantly underrepresented in the most distant control arm. This result is consistent with weak attraction to this low concentration of the target compound.

## Discussion

Our results clearly demonstrate that *E. fetida* are attracted by olfactory cues associated with *G candidum*, and thus complement previous reports that earthworms are able to actively search for food sources [20]. We furthermore identified two specific compounds from the filtrate of *G. candidum* colonies that exhibit significant attraction for *E. fetida*, the esters ethyl pentanoate and ethyl hexanoate. To the best of our knowledge, no previous studies have identified specific olfactory cues used by earthworms. In nematodes, attraction has been shown for unidentified olfactory cues deriving from insect larvae [32], and for several specific chemical compounds, including diacetyl, (E)-ß-caryophyllene, isobutanol [14], [15]. In *C. elegans*, chemiotaxis to volatiles were observed for at least 50 compounds, and specific neurons and genes involved for these responses have been described [1], [33]. The perception of volatile odorants by *E. fetida* may also involve some specific corporal receptors associated with neurons. Indeed, earthworms are known to have chemoreceptors, principally on the prostonium or on the buccal epithelium that are associated with the nervous system and more particularly with axons and dendrites [18]. The foraging strategy of *E. fetida* may also bear similarity to social strains of *C. elegans*, as these worms have been observed to aggregate in areas where bacteria are numerous [34] and there is some evidence for coordinated movement in *E. fetida* [19].

The two esters we found to be attractive to *E. fetida* have previously been shown to function as cues for insects in other systems. Ethyl pentanoate has an attractant activity for the dung beetle, *Pachylomerus femoralis* [35], and ethyl hexanoate, in combination with 1,8-cineole and hexanol, attracts the Mexican fruit fly, *Anastrepha ludens*, to fermenting, immature fruit of yellow chapote [36]. The latter compound also stimulates upwind flight of the lepidopteran, *Ectomyelois ceratoniae* [37].

Among the molecules we identified from *G. candidum* filtrate, ethyl propionate, ethyl acetate, 3-methylbutan-1-ol, 2-methylbutan-1-ol and 2-methylpropanol have previously been found in the volatile profile of *G. candidum* and other microorganisms [38], [39]. The formation of 2-methylpropanol, 2-methylbutanol, and 3-methylbutanol by *G. candidum* almost certainly involves deamination of glutamic and aspartic acids and of leucine, phenylalanine and methionine, which are commonly found in fungi [40], [41]. Two other molecules emitted by *G. candidum* filtrate, hexanoic acid ethyl ester and 3-octanone, were previously identified as volatiles from the fungi *Aspergillus candidus* [39]. Different strains of lactic acid bacteria are able to synthesize ethyl ester from 2 to 10 carbon atoms, mainly ethyl hexanoate [42]. The hydrolysis products of *G. candidum* lipases may be the precursors of various volatile compounds such as alcohols, methyl ketones and esters [40].

Earthworm attraction to chemical cues associated with food has potential application for the development of techniques for the extraction and sampling of earthworms, for example in vermicomposting. Other behavioral techniques have previously been employed for such purposes, including heat extraction, electrical extraction, and mechanical vibration [18], [43], [44], and chemical extraction methods using natural repellents or irritants, like formalin, mustard extract, exotic-plant extracts have been reported [45], [46], [47], [48], [49]. Because they are based on attraction rather than repulsion, the esters presented above may have advantages over the existing chemical methods (e.g., efficacy when applied at low concentrations and on restricted spatial scales) but confirming this will require further study.

In conclusion, this study provides the first documentation of specific olfactory cues involved in annelid foraging. Microbiota are key producers of volatile compounds in soil ecosystems [24] as well as major components of earthworm diets [20]. Thus, further elucidation of the mechanisms by which earthworms perceive and respond to olfactory cues will enhance our understanding of the ecology of soil ecosystems, in which earthworms play a tremendously important role in temperate regions. Furthermore, exploration of earthworm olfaction will help us to understand how these animals orient themselves and coordinate their behavior. For example, it has previously been suggested that chemical cues are involved in earthworms [18], [50], and there is some evidence that *L. terrestris* follows mucus trails to find its partner [51], but the role of volatile perception in such interactions remains to be documented. Finally, as noted above, such work has potential implications for the development of techniques for the extraction and sampling of earthworms in vermicomposting and other applied settings.
